# Marburg virus: Africa’s deadly intruder exposed

**DOI:** 10.1097/MS9.0000000000001227

**Published:** 2023-09-04

**Authors:** Maryam Shahzad, Sean K. Shaeen, Abdullah Malikzai

**Affiliations:** a Dow University of Health Sciences, Karachi, Pakistan; b Kabul University of Medical Sciences, Kabul, Afghanistan

*Dear Editor*,

On the 13th of February 2023, Equatorial Guinea confirmed its first case of the Marburg virus (MARV)^1^. Since then, a total of 17 laboratory-confirmed cases of Marburg virus disease (MVD) and 23 probable cases have been reported as of the 1st of May. The most affected district is Bata in Littoral province with 11 laboratory-confirmed MVD cases reported. All the probable cases have expired. Among the laboratory-confirmed cases, 12 deaths were recorded^1^.

No current cases have been reported by the MARV treatment center as of the latest patient release on 26 April 2023. This brings the count of total survivors to 4, since the official declaration of the outbreak.

The MARV, is closely related to the Ebola virus, which belongs to the *Filoviridae* family (filovirus)^2^. The virus is transmitted to humans via inhalation of tainted bat excreta or contact with body fluid from already infected individuals. After the initial contact, MARV enters the body, reproduces, and spreads, causing a clinical condition that includes fever, malaise, myalgia, and problems with blood coagulation^3^. In many instances, these symptoms proceed to shock, multiple organ failure, and severe hemorrhagic fever which contributes to its significant fatality ratio of 88% thus making it a potential threat to public health and safety^4^.

Through a multitude of combined efforts between the Equatorial Guinea government and the WHO have helped curb the spread of the MARV. A regional public health emergency operation center was established in Bata. Furthermore, with the help of the Center for Disease Control, a laboratory with RT-PCR (reverse transcription polymerase chain reaction) capacities was established for MVD diagnostic training as well as the training of local staff^1^. The WHO is also involved in overseeing the collection and transportation of samples in order to ensure quality and timely testing of samples^1^. Additionally in tandem with the Ministry of Health, the WHO also set up an operational MVD alert and dispatch center that focuses on the training and support of staff on the supervision of surveillance activities^1^. These surveillance activities include case investigation, contact tracing, and coordinating with healthcare facilities for active surveillance^1^. Lastly, the burial of suspected cases also constitutes a risk for the spread of MVD themselves^1^. Therefore, the WHO has dispatched and established burial teams in Bata and Ebibeyin to monitor the burial practices of suspected cases of MVD^1^.

The WHO has classified the MVD outbreak risk as very high at the national level, high at the sub-regional level, moderate at the regional level, and low at the global level, therefore Equatorial Guinea has to further strengthen its advances in order to control the spread of the virus^1^. Firstly, fluid movement at the borders constitutes the greatest risk for spread of the MVD at national level. High porosity of local borders and constant migration of people makes it difficult to trace and keep track of contacts which leads to the spread of the MARV in other parts of Africa. Lack of border security combined with fluid movements between Ebebiyin and Nsock Nsomo districts (Equatorial Guinea), Cameroon, and Gabon especially constitute the risk of cross-border spread^4^. The last cases occurred in Bata, a metropolitan with both an airport as well as a seaport which can lead to further spread of the virus to other countries due to weak surveillance^1^. The Government of Equatorial Guinea needs to strengthen surveillance in the country especially at points of entry in the affected areas through alert management, case investigation, contact listing, tracing and follow-up, and active case search^1^. The MARV also has the potential to spread globally therefore, international as well as local travel needs to be restricted in order to prevent this.

Not only Equatorial Guinea, but African countries in general need to enhance preparedness for future outbreak. African countries should prioritize the recruitment of additional healthcare professionals to address the existing shortage of workers^5^. To address the shortage, it is important for the African government to increase the recruitment and training of doctors, nurses, and healthcare professionals. It would also be beneficial to train local workers, particularly those from the most affected communities, as survivors who are most likely immune can play a vital role in raising awareness about isolation and early diagnosis^5^. This approach not only reduces the gap between community members but also promotes community engagement and connection. Additionally, the issue of ineffective healthcare financing often results in exorbitant costs that prevent the general public from accessing proper healthcare. While medical insurance programs exist, they are not designed to assist the poor, thus excluding them from crucial coverage^6^. Considering that the poor are the most susceptible during disease outbreaks, it is imperative for the government to create and implement social health insurance programs specifically aimed at helping the impoverished population in order to make healthcare more accessible and attainable for the impoverished.

The MARV disease treatment typically involves oral rehydration and supportive care. However, there are promising advancements in the form of new vaccines. One such vaccine in development is the VSV-MARV, a recombinant vesicular stomatitis virus-based vaccine that expresses the MARV glycoprotein. Animal models have demonstrated its rapid protection against MVD^7^. Additionally, researchers are working on post-exposure therapies, including MARV-specific monoclonal antibodies (mAbs) and small-molecule antivirals. In a non-human primate model, the combination of the monoclonal antibody MR186YTE and the antiviral remdesivir proved highly effective in eliminating the virus^8^.

In summary, Equatorial Guinea with help from numerous healthcare organizations effectively handled the MARV outbreak as no additional cases have been reported. Although there are many contributing factors to this success, it is also imperative to learn from this by taking note of aspects where the country was lacking as a way to improve its ability to handle future outbreaks. Therefore, there is still a need for the country to focus on enhancing its healthcare system framework, work on its tracing abilities, enhance border security, and awareness to help improve healthcare in general as well as be prepared for future outbreaks.

## Ethical approval

Ethics approval was not required for this short communication article.

## Consent

Not required for this short communication article.

## Sources of funding

Not applicable.

## Author contribution

All authors equally contributed.

## Conflicts of interest disclosure

The authors declare that they have no conflicts of interest.

## Research registration unique identifying number (UIN)

Not applicable.

## Guarantor

Abdullah Malikzai.

## Data availability statement

Not applicable.

## Provenance and peer review

Not applicable.
